# Improving the Quality of Life of Patients with an Underactive Thyroid Through mHealth: A Patient-Centered Approach

**DOI:** 10.1089/whr.2021.0010

**Published:** 2021-06-28

**Authors:** Vedrana Högqvist Tabor, Mikael Högqvist Tabor, Sarai Keestra, Jean-Etienne Parrot, Alexandra Alvergne

**Affiliations:** ^1^ BOOST Thyroid by VLM Health UG, Berlin, Germany.; ^2^ School of Anthropology and Museum Ethnography, University of Oxford, Oxford, United Kingdom.; ^3^ Amsterdam UMC, University of Amsterdam, Amsterdam, The Netherlands.; ^4^ ISEM, Université de Montpellier, CNRS, IRD, EPHE, Montpellier, France.

**Keywords:** disease self-management, female health, mHealth, patient empowerment, mixed methods, quality of life

## Abstract

***Background:*** Three hundred fifty million people worldwide suffer from underactive thyroid conditions, which can lead to infertility, obesity, heart disease, and impaired mental health when poorly managed. Although mobile health (mHealth) applications can be a useful solution for self-managing one's condition, the impact of digital solutions for improving the health of thyroid patients remains unknown.

***Methods:*** We used a mixed methods analysis to assess the ways in which a digital approach might benefit thyroid patients. A cross-sectional study was conducted among users of BOOST Thyroid, an mHealth application for patients with an underactive thyroid. We collected data using a modified Short Form 36 Health Survey Questionnaire to measure the impact of in the app on participants' perceived health and quality of life. Participants were asked to (1) score their quality of life before and after using the app, and (2) describe whether and how using the app helped them.

***Results:*** We enrolled 406 users (380 females and 26 males), aged 18–78 years. Most participants (95.8%) reported using the app was helpful; of which 68% reported it improved their quality of life and 70.8% reported it had a positive impact on their health. Participants who found the app useful experienced less symptoms and a lower intensity of remaining symptoms. A key factor reported by these participants as helping with managing their health is the information provided in the app.

***Conclusions:*** The results support the idea that a patient-centered treatment would benefit from including mHealth tools for a daily self-management of underactive thyroid condition, as it can increase health literacy and improve both one's health status and quality of life.

## Introduction

An underactive thyroid is the most common endocrine disorder worldwide affecting an estimated 350 million people,^1–4^ predominantly females.^5^ Patients with an underactive thyroid need continuous care, as poor health management leads to several health complications, including obesity, depressive spectrum disorders, and increased risk of female infertility.^2,4,6–12^

Yet, thyroid conditions are notoriously hard to manage. First, symptoms are diverse in their intensity and duration.^13^ Consequently, universally prescribed l-thyroxine monotherapy does not have the desired effects in up to 15% of patients, a cause for much annoyance and discomfort as well as a broken patient–doctor relationship.^14^ Second, there are gender differences in accessibility to health information and health services, which, given thyroid conditions predominantly affect females, negatively impact thyroid health management.^15,16^ Given this, a new patient-centered approach using mobile health (mHealth) technologies might be promising for delivering a more personalized care to thyroid health. Yet, to date, it is unknown whether the use of mHealth can help address barriers to effective thyroid health management.

The prevailing “one size fits all” approach to thyroid health leads to poor thyroid health management at the individual level. The underlying issues are (1) *uniform treatments*: the treatment choices in thyroid patients rely on generalized guidelines^17–21^; (2) *limited patients' understanding of thyroid condition:* because of a lack of accessible evidence-based resources on thyroid health, patients are often unsure if the symptoms they experience relate to their thyroid condition^19^; (3) *lack of frequent medical follow-ups based on patients' needs*: once patients have been diagnosed and their medication dose established, follow-up checkups are, on average, only once a year.

This frequency is independent of individual patient's symptoms and changes to their health with thyroid stimulating hormone (TSH) values often used as a sole indicator of the thyroid status and well-being^19,22^ The exception to this approach is in the case of pregnancy, when more regular checkups and therapy adjustments protocols are followed.^19–21^ These issues have led medical health care professionals, patients, and researchers alike, to call for a patient-centered approach to the treatment of thyroid conditions considering the unique experiences and needs of individuals.^19,23^ Such an approach requires taking one's symptoms, lifestyle, overall well-being, additional comorbidities, and life stage into consideration when making medical decisions.

To overcome the barriers to adequate thyroid health management at an individual level, mHealth technology offers a promising avenue. To date, mHealth has proven beneficial for managing chronic health conditions, including diabetes,^20,24–27^ chronic lung disease,^27–29^ cardiovascular disease,^20^ chronic disease,^27^ and mental health disorders.^30,31^ However, its potential for managing thyroid health remains unknown although there are various reasons why mHealth tools can benefit thyroid patients.

Firstly, mHealth is accessible to a large population and it has been suggested that using a digital solution can help patients understand their symptoms, negotiate medical encounters, and provide practical support between medical appointments to maintain good health.^32^ Secondly, mHealth solutions can assist public health goals when acting as a companion to therapy that can be utilized by a large population of patients.^27,30,33–35^ Thirdly by providing information and answers to triaged patient questions, mHealth tools can help reduce the cost of health care by saving physicians' time.^27,30,36–38^ Finally, mHealth is of interest to researchers because it enables collecting real-time and granular symptom data across a large population for extensive periods of time.^34^ Such novel data are critical to increasing knowledge on under-researched health conditions, particularly to advance the agenda on female health.^39^ Although gender plays an increasingly well-recognized role in creating and implementing health policies, especially for the treatment of chronic conditions,^40,41^ sex-specific data on chronic health conditions are often missing^42–45^ and, therefore, not taken in account by policy makers.

This study aims to investigate the ways in which an mHealth approach benefits thyroid patients. To do this, we conducted a cross-sectional study among users of the BOOST Thyroid app. First, we used an adapted version of the Short Form 36 Health Survey Questionnaire, a standard for assessing health: physical functioning, role physical, bodily pain, general health, vitality, social functioning, role emotional, and mental health to quantify whether, how and the extent to which information, symptom logging, and analytics features provided in the app were associated with an increase in (1) perceived health, (2) perceived quality of life, and (3) positive interactions between patients and health care professionals.^46,47^ Second, to capture the voices of participants, an open-ended question was included to collect qualitative data on the experiences of using the app for managing thyroid condition.

The survey was distributed to users through the app and was run for 3 months. In addition, we analyzed de-identified data on users' symptoms throughout the first 3 months of the app usage, to get a long-term insight on symptom improvement through app usage. Finally, we compared TSH and body mass index (BMI) levels between the users of the app and the means reported in the literature to avoid introducing bias from differences in TSH and BMI. We found that using the app is helpful to the majority of users. Among users who find the app useful, information came out as the most important feature to improve one's health and quality of life. The study has implications for understanding how mHealth can help manage thyroid conditions.

## Methods

### Study design, population, and recruitment

We recruited users of the BOOST Thyroid app, 1 of the 10 thyroid health tracking apps available in the Apple Store for iPhones. The BOOST Thyroid app was launched in 2017 globally, with an intended use for health management of patients with an underactive thyroid. The app provides (1) logging of symptoms on a 5-point intensity scale, medication adherence, and laboratory tests; (2) information on symptoms, medication, supplements, and on different aspects of thyroid condition; (3) analytics of symptoms in the past 7–365 days; (4) algorithm calculating the basal metabolic rate of each individual; (5) PDF report on symptoms. The app has at least one software update a month, and is available in English, German, and Spanish (Supplementary Fig. S1). The BOOST Thyroid app versions used during the time of the survey were 20.3.1.–20.6.1. The app underwent a total of two updates, with the addition of logging of thyroidectomy diagnosis and new articles (on COVID-19 pandemics, thyroidectomy, and weight management) to the database of 80 already existing articles. The users of the app are 86.7% females and 13.3% males, with age range from 18 to 65+ years (Supplementary Table S2 and Supplementary Fig. S2A, B).

The study received approval from the Ethics Committee of University of Oxford, SAME_C1A_20_001. The survey was distributed to all users through the BOOST Thyroid app home screen (the first screen users come to when opening the app, Supplementary Fig. S1A). Participants had used the app between 7 days and 2 years before taking part in the survey. Survey responses were collected from March 2020 to July 2020, a period set *a priori*. Users agreed to (1) general terms of service before they start using the app, agreeing for their anonymized data to be used in research; (2) participate in the survey and were informed on how to withdraw their data. No personally identifiable data were collected for this study. The data were stored on a secure server and kept separate from any personal identifiers in accordance with the EU General Data Protection Regulation.^31^

### Data

Participants were first asked whether the mHealth solution has helped them “in general.” Only participants who indicated that the app is useful continued to participate in the survey to draw upon the positive experiences of mHealth solutions for underactive thyroid. To assess how the app is helpful to users, we included binary, single-choice, and multiple-choice questions (Q1–8, Supplementary Table S1). To capture participants' experiences in context, the survey also included the free-text question “Can you tell us 3 ways the BOOST Thyroid app has influenced your health?” To evaluate users' quality of life before and after using the app, we used a modified version of the Short Form 36 Health Survey Questionnaire.^46,47^ We included specific and appropriate questions relating to the health and the quality of life of individuals with an underactive thyroid, reflecting the most common symptoms and the most common day-to-day life problems based on the scientific literature (Refs.^21,22,44,48^ and Supplementary Table S1). Some questions are measured on a 5-point Likert scale; others are measured on a binary scale or allow for multiple answers (Supplementary Table S1). The resulting questionnaire resulted in a maximum of 19 questions, asked depending on users' answers. Finally, data on symptoms, age, BMI, and TSH values previously entered in the app by users were paired with survey data.

In addition, we analyzed anonymized data of five (5) most tracked symptoms from 115 users at day 0 (first day of the app usage) and day 90 of app usage. These five symptoms correspond to additional questions present in ThyPro™ questionnaire, specific for all the thyroid conditions (Ref.^49^ and Supplementary Fig. S3).

### Statistical analysis

Raw data were analyzed and drawn using PrismGraph software. Categorical variables were summarized as counts and percentage, whereas numerical variables were summarized as mean and SD (or median and IQR). We first compared participants with the larger pool of anonymized app users with regard to age, BMI, and TSH levels. Second, to compare participants' self-reported quality of life before and after using the app, two-sided chi square tests for comparing proportions of categorical variables and nominal symmetry tests for paired data were performed using the R software and the package “rcompanion.”^50,51^

### Analysis of qualitative data

The qualitative data were analyzed using NVivo12.^52^ Participants' quotes were reviewed and tagged with “codes” to extract themes from the data independently by two members of the research team trained in qualitative analysis. As more data were reviewed, codes were grouped into concepts and a concept map was drawn to depict relationships between concepts and themes. Multiple readings were conducted and alternative explanations of the data explored to develop a robust interpretation of the findings.

## Results

### Description of participants

A total of 406 users participated in the survey (380 women and 26 men, response rate = 77.1%). Participants of this cross-sectional study are broadly representative of the BOOST Thyroid users' population in terms of age, age at diagnosis, TSH values, and BMI (Supplementary Fig. S2 and Supplementary Table S2). Survey respondents are mainly females (93%), with age distribution peaking between ages 25 and 54 years, a pattern similar to the majority of app users and to the distribution within the global patient population (female to male ratio 10:1 and diagnosed between their 30s and 50s^5^ and Supplementary Fig. S2A, B and Supplementary Table S2). TSH values also do not vary significantly between the app users and survey respondents. Although participants were in a range of 0.6–4.2 mIU/L, a range less broad than that of other users of the app, the difference is not statistically significant, all the values are within the reference ranges applied in the clinical practice worldwide (Supplementary Fig. S2D and Supplementary Table S2 and Ref.^53^). Finally, BMI was ranging between 27 and 31 kg/m^2^ for the general the population of app users (mean 27.6 kg/m^2^) and between 26 and 30 kg/m^2^ for the survey respondents (mean 26.3 kg/m^2^) (Supplementary Fig. S2D and Supplementary Table S2).

### Quantitative findings

Out of the participants who answered, 95.8% indicated that using the mHealth solution has helped them “in general.” Out of the 4.2% of participants who reported that the app was not helpful, 52.4% responded that they already knew the information shown in the app. Only participants who have indicated that the app is useful continued to participate in the survey.

#### Increasing health literacy through information on thyroid health helps patients understanding their symptoms

**FIG. 1.**
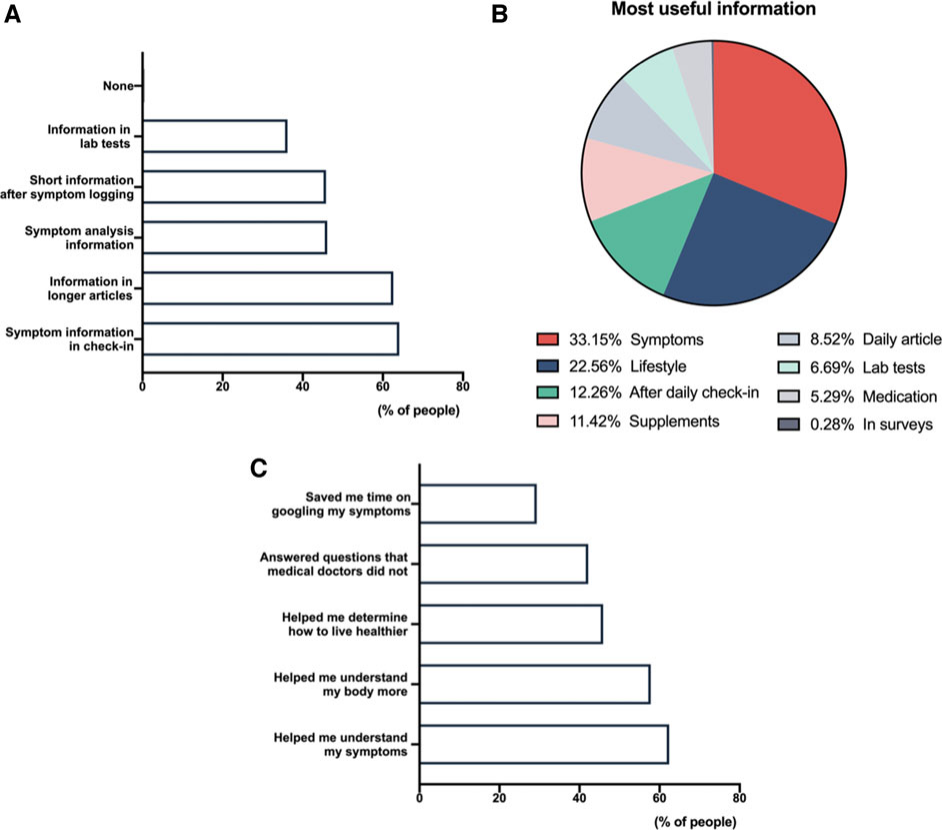
Information benefit provided through BOOST Thyroid app. **(A)** Useful types of information. **(B)** The most useful type of information. **(C)** Exact benefit of the information.

App users indicated different parts of the app were of help to them, with, 62% of the survey participants indicated that information within the app helped them understand their symptoms better (Fig. 1C). The main sources of information participants reported to be beneficial were informational text provided in every tracking category (helpful for 64.1% of participants), information contained in longer articles (62.6%), information on symptom analytics (46.1%), short information after symptom logging (45.8%), and information provided on the laboratory test logging screen (36.2%) (Fig. 1A and Supplementary Fig. S1B). Out of those, the most useful information concerns symptoms (31%) and lifestyle (diet and exercise) (22.6%) (Fig. 1B).

#### Using mHealth improves patient–doctor interaction

**FIG. 2.**
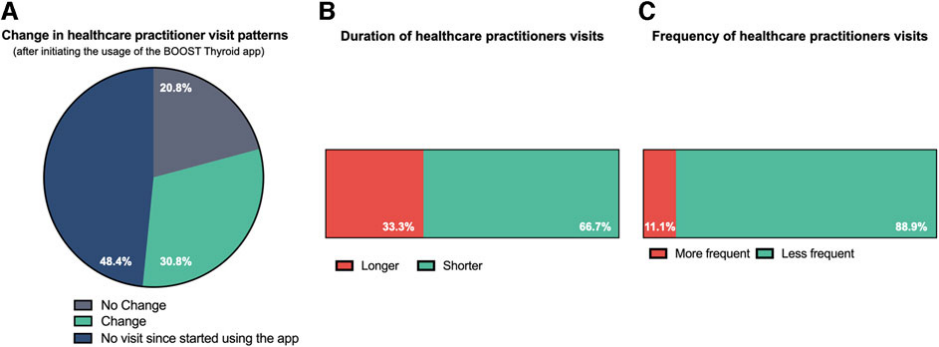
Changes in health care practitioners visit patterns after using the BOOST Thyroid app. **(A)** Assessment of change in the visitation patterns before and after using the app. **(B)** Changes in the duration of individual visits to health care practitioners. **(C)** Changes in the frequency of visits to health care practitioners.

Next, in the population of patients who reported to have benefited from using the app (95.8% of survey respondents), we evaluated whether using a digital health tool can improve patient–doctor interactions. We found that doctor visits did change in frequency and duration after starting using the app for 30.8% of respondents, while 48.4% of respondents did not visit a doctor since they started using the app. The survey was partly conducted during COVID-19 restrictions, which might have impacted the possibility of visiting doctors. For 20.8% of participants, doctor visits frequency and duration did not change since they started using the app (Fig. 2A). For people reporting changes, visits were shorter for 58.3% and less frequent for 88.9% of respondents (Fig. 2B, C).

#### Using mHealth specifically improves patients' perceived health and quality of life

**FIG. 3.**
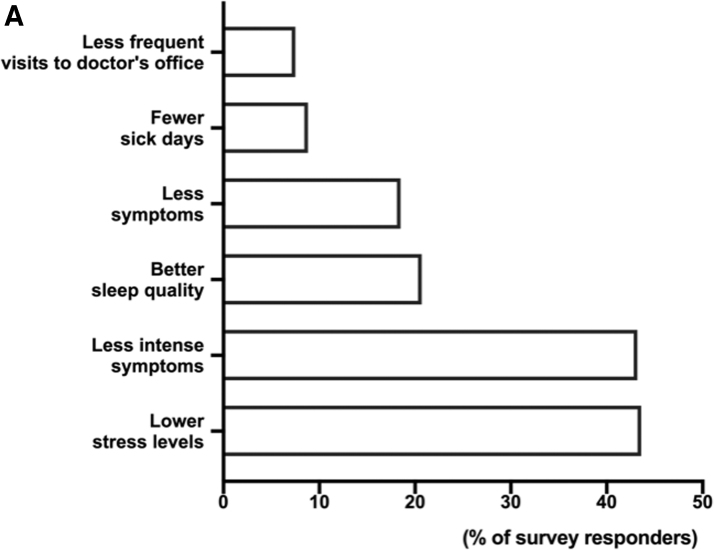
Benefit of the BOOST Thyroid app to everyday life of the app user. **(A)** Assessment of six main benefits of the BOOST Thyroid app.

We found that 70.8% of participants who found it helpful to use an mHealth solution (95.4%) reported that using the app has had a direct positive impact on their health—18.1% had fewer symptoms and 42.3% had less intense symptoms (Fig. 3A).

**FIG. 4.**
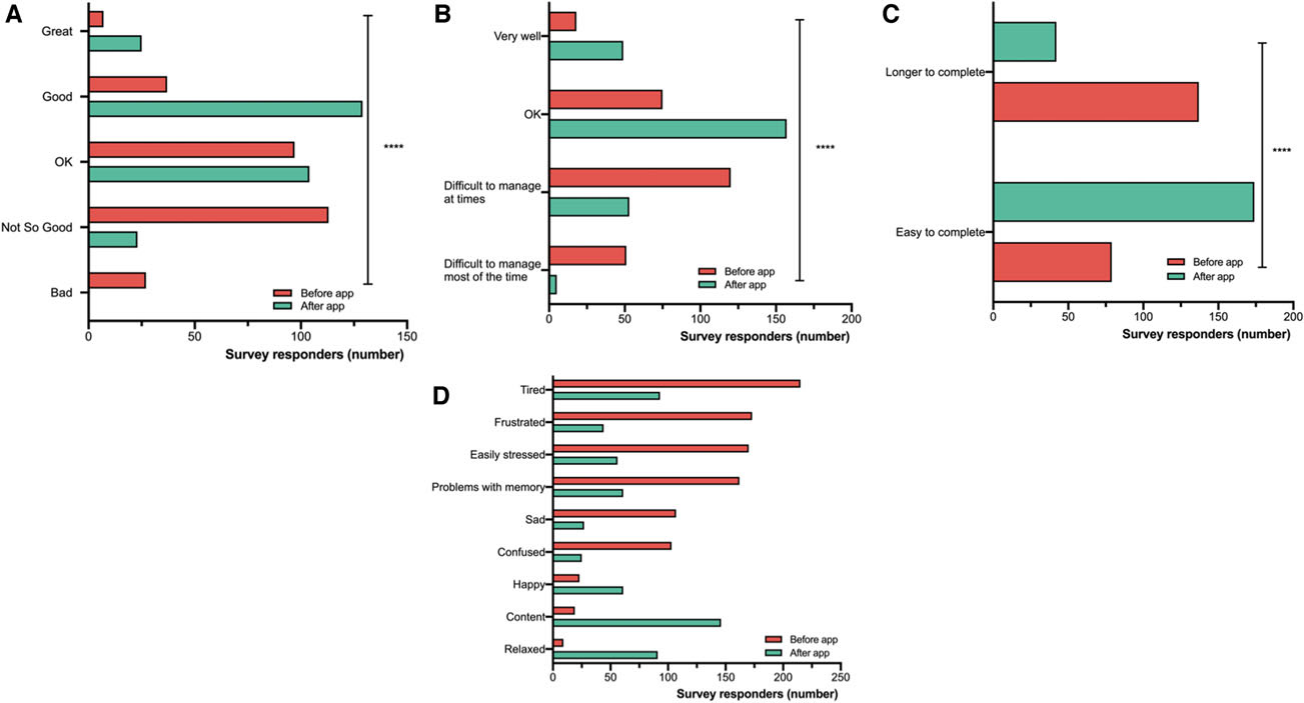
Comparison of the life quality before and after using the BOOST Thyroid app. **(A)** Assessment of general feeling on a 5-point intensity scale (Great, Good, OK, Not so Good, and Bad) before and after using the app (*p* < 0.001). **(B)** Assessment of managing life tasks before and after using the app (*p* < 0.001). **(C)** Assessment of easiness of completing tasks before and after using the app (*p* < 0.001). **(D)** Assessment of general feeling before and after using the app (nine options in a multiple select answer), (*p* < 0.001).

We assessed on a 5-point Likert intensity scale (great, good, ok, not so good, and bad) how participants felt before and after using the app. Before using the app, 49.8% felt “bad” or “not so good,” 34.5% felt “ok,” and 16.2% felt “good” or “great.” After using the app, 8.2% of respondents still felt “bad” or “not so good,” 37% of respondents felt “ok” and 54.8% felt “good” or “great” (Fig. 4A). Thus, people reported feeling better after using the app (nominal symmetry test, *p* < 0.001). Difficulties with managing life and work decreased nearly threefold from 64.8% to 22% of respondents after using the app (Fig. 4B, nominal symmetry test, *p* < 0.001). The ability to easily complete tasks was increased twofold: 36.6% before using the app vs. 80.6% after (Fig. 4C, nominal symmetry test, *p* < 0.001).

The feelings most frequently reported by the participants before using the app were being tired, frustrated, and easily stressed. After using the app, the feelings most frequently reported were content, relaxed, and tired (Fig. 4D and Supplementary Table S3).

### Qualitative findings

**FIG. 5.**
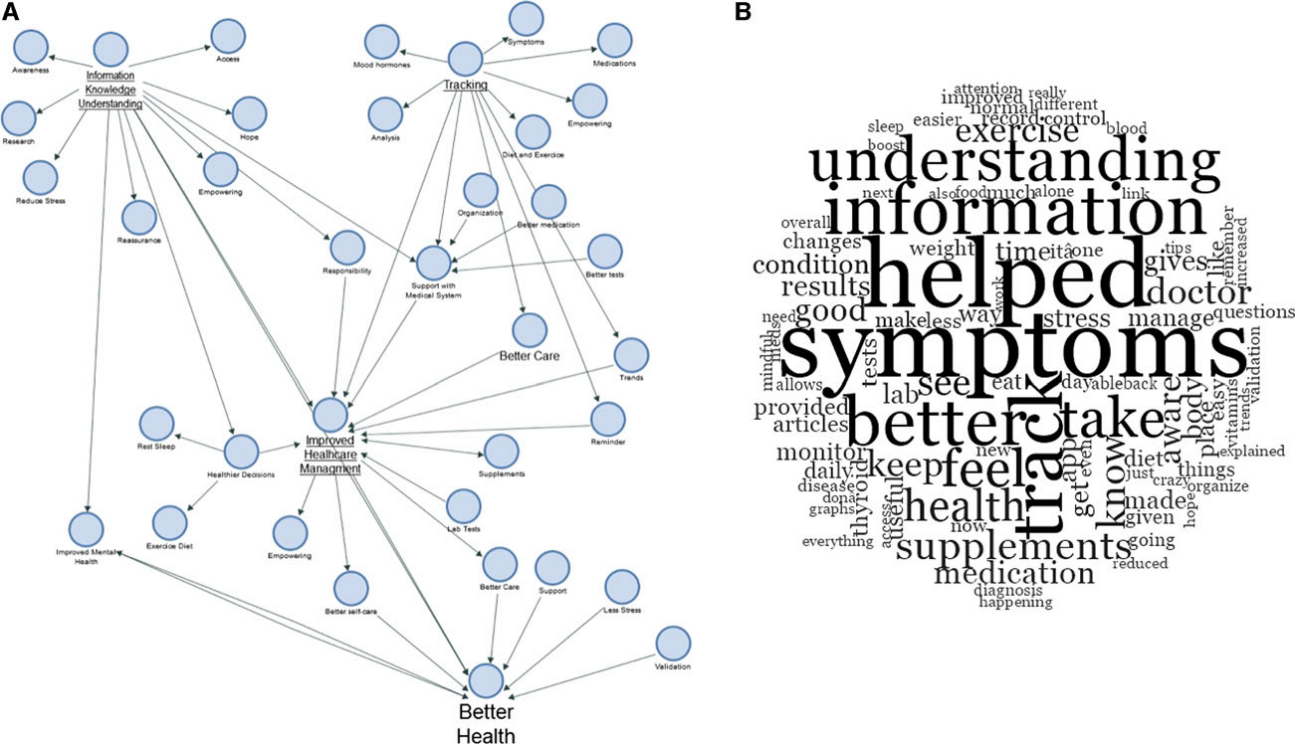
Themes and reflections of underactive thyroid patients after using the BOOST Thyroid app. **(A)** A concept map based on the themes emerging from the qualitative data analysis. **(B)** A word cloud of most frequent words used by survey respondents.

Drawing on participant's own words, multiple features of the app contributed to help managing their health, including the research driven and easily accessible information provided, the ability to track symptoms, medications and other aspects of daily life, as well as reminders (Supplementary Tables S4 and S5). First, using the app led to increased awareness, which in turn led to better medical tests and more appropriate medication regime. Participants reported that using the app enabled them to recognize trends and patterns in well-being as well as notice when symptoms were getting better or worse. One participant wrote that using the app gave them “*more awareness of my own body and proof that issues such as symptoms are not just in my head.*” Second, using the app led to better care of the self, healthier decisions, and improved mental health. Indeed, users described feelings of loneliness and “*being crazy*” before using the app, issues that were then mitigated after using the app, which provided them with validation, reassurance, and control. Third, users reported that tracking symptoms enabled them to share experiences of health and well-being for longer periods of time with their general practitioners and endocrinologists, thereby enriching health care interactions. For instance, participants reported that that they could better organize their laboratory results by making use of the app, so they could see changes for the long time and share this information during their medical visits. Using the app made the doctor's visits of some participants “*more focused*,” leading to “*better conversations and follow-up questions with my doctors.*” Finally, users reported that using the app led them to make better lifestyle decisions and that they felt empowered to take additional steps to improve their health condition outside of medication alone, such as changing sleep patterns, diet, or exercise regimes. Overall, participant responses suggested that using the app can lead to a perception of increased empowerment and feeling of control, improved health care management, and better health (Fig. 5).

## Discussion

This article set out to investigate whether and how mHealth can improve the health and quality of life of patients with an underactive thyroid. Recent data suggest that not all patients with an underactive thyroid should be treated in the same way and a patient-centered treatment approach is needed.^19,22,27,30,33–35^ Indeed, thyroid patients experience a plethora of different symptoms with varying intensities, reducing their quality of life and work capabilities in various ways.^48^ To deliver personalized patient-centered care, which is hard to accomplish, an mHealth approach that leans on Big Data and Deep Learning might offer a promising avenue as it can provide specific insights for each patient.^54–56^ By analyzing responses from users of the BOOST Thyroid app, which indicated they found using an mHealth solution beneficial, we found that using a digital tool specifically helps these patients to (1) reduce symptom severity and the number of symptoms experienced, (2) improve day-to-day functioning and ease of daily tasks completion; (3) increase the feeling of being happier/more accomplished, (4) reduce the number of sick days, (5) improve patient–doctor conversation. The findings reveal a largely untapped potential for mHealth tools to be used for a more individualized approach to managing an underactive thyroid.

According to the majority (95.8%) of participants, using the BOOST Thyroid app had a positive impact on their quality of life and for 67.8% of survey participants, the app had a direct positive effect on their health. In our sample comprising respondents who downloaded the app and found using the app to be beneficial, individuals reported an increase in their overall well-being and their ease of completing everyday tasks, which suggests that patients can improve day-to-day functioning and work capacity by using digital health. This is specifically valuable considering that the decrease in productivity caused by chronic health problems, such as thyroid dysfunction, puts a major burden on the workforce and the economy.^57^ Second, we found that using digital health improved patient–doctor interactions. This is important as the burden of managing chronic conditions reported by health care professional is currently exceeding their time capacities.^58^ Participants indicated that detailed symptom data were useful to share with medical practitioners, as some users reported that it led them to have better medical outcomes. A more efficient doctor visit through a concise presentation of symptoms and specific questions of interest, with no information loss, can save time and lead to improved shared decision-making. Third, for some participants, using the app led to better care of themselves, encouraging lifestyle changes that aid in the management of thyroid health. Information from the scientific literature enabled users to better understand and discover symptoms. Together with the possibility of self-tracking, users said they (1) have a “*better understanding of results*,” “*better understanding of my symptoms*,” “*more awareness of my own body*”; (2) are “*able to see trends arise from all the data collected, which better enabled me to get the correct medication dose*”; and (3) “*feel better informed*,” “*have better conversations and follow-up questions with my doctors*,” and “*improved my mental health*.” In turn, improved health care management, combined with the feeling of being “*validated*,” that is, that one's experience is being understood and accepted by others, led users to feel empowered to self-manage their health.

### Limitations

As a cross-sectional study of current users of a digital health app, this study has several limitations. First, real life studies are designed to assess the effectiveness of an intervention in normal everyday life circumstances. Results of such studies have the potential to be applied to a larger population, with a limitation of mHealth approach introducing a bias toward the population ready to use mHealth apps to manage their conditions. We are particularly aware that some patient groups, especially older adults, may face challenges when it comes to adopting new technologies. Indeed, one in three of internet users >65 years report that they have little to no confidence in their ability to use electronic devices to perform online tasks,^59^ which may be a barrier to adopting a digital approach toward greater patient-centered care in thyroid health. To allow for a digitalized approach, mHealth needs to be adjusted to accommodate for the older audience, and pay attention to age, gender, disability, and cultural accessibility: starting with enabling people with visibility impairment to hear voiceovers or have larger fonts, and ensuring quick support and manuals, where people can be helped with the app setup. These services are already covered by most health apps, including the BOOST Thyroid app. Future software iterations of mHealth should focus on inclusivity with a specific focus on providing users with the skills and competencies to fruitfully engage in a digital approach as well. It should be noted however that in 2020 there were 3.6 billion smartphone users worldwide, and 69% of smartphone users reported using health apps, this massive adoption to smartphone distributed health, combined with big advancements in technology will lead to mHealth becoming a dominant approach to managing own health in the years to come.^60^

Another limitation of this study is that it relies on the accuracy of self-reported data, and there is a limited way to control the accuracy of answers. In addition, this study did not exclude individuals with other comorbidities that might impact perceptions of health and well-being. Furthermore, the experiences of health care professionals in interacting with patients using mHealth were not captured in this study, limiting the measured effect on the patient–doctor interaction to the perception of patients only. Yet, data from a previous market research done by the BOOST Thyroid app (not shown here) indicates that health care professionals find coherent symptom presentation provided by the mHealth solution useful for their interaction with patients and the shared decision-making process. Finally, in this study, we have not included additional biomarkers of thyroid function beyond TSH, which (although globally accepted as the first indicator of an underactive thyroid) is not usually sufficient to fully determine thyroid status.^17^

It could be argued that health education alone, rather than health education through the app, is what drives our results. Indeed, the improvement observed in the participant cohort of current users of the app, who indicated the mHealth solution to be helpful, may be a result of the type of information shown to people and the medium through which the information was disseminated, which allowed for more personalized approach. Although answering this question would require a randomized controlled trial, there are various reasons why augmenting health education with an app might prove useful. For instance, through displaying appropriate articles of interest and in-depth analytics and interpretation of symptoms, this type of information may offer more salience for patients interested in charting and understanding their symptoms, when compared with the traditional approach of leaflet distribution as an educational intervention. Additional advantages of mHealth compared with leaflet distribution may be that (1) apps can be distributed to millions of people globally in a short period of time; (2) they can be quickly updated to respond to the changing patient needs (as with the SARS-CoV-2 pandemic), (3) they can provide a digital vault for all the patients information, which can be easily shared with their physicians, (4) they provide opportunity for interactions between patients and health professionals in times when access to health care is restricted, such as during the COVID-19 lockdown. A randomized controlled trial involving newly diagnosed hypothyroid patients should ideally be run, comparing the mHealth approach with other educational interventions catered for patients with an underactive thyroid.

Finally, for the purpose of this study we opted to use a modified version of SF-36, a general quality of life questionnaire used worldwide, as a first step of assessing the benefit of using an mHealth solution to the quality of life of thyroid patients who indicated that they found the digital health app useful. Although there are other more specific thyroid questionnaire forms, such as ThyPro,^49^ which would have been a more appropriate choice if we would have tested the entire spectrum of thyroid diseases, as we focused on hypothyroidism only, we would have had to exclude certain parts of the questionnaire by default. Second, ThyPro is specifically adjusted for use in different countries, making it more challenging for a global rollout to the audience of a digital app with users in >70 countries worldwide. Instead, for a majority of the questions shown in ThyPro questionnaire relevant to hypothyroid conditions, we have a counterpart from the tracking data, including (1) Have you been sensitive to cold? (2) Have you felt energetic? (3) Have you had difficulties remembering? (4) Have you had slow or unclear thinking? (5) Have you felt afraid or anxious? (Supplementary Fig. S3).

More than four-fifths of the world's population has access to a mobile phone with increasingly powerful technical capacities, making mHealth a potentially desirable and global solution to help with the self-management of chronic conditions.^61^ Yet there remain some barriers to applying mHealth more fully, including doctor's resistance,^62,63^ high data entry burden for patients, and potential loss of interest in the long term.^64^ The lack of access to affordable internet in some localities might cause reduced availability of mHealth tools and free health information.^65,66^ Furthermore, some might argue that the quality of the information when designing and building mHealth solutions is not always appropriate, and scientifically accurate. However, the data protection regulations and laws spanning the EU, United States, Canada, and Brazil give a lot of power back to users of these technologies in terms of data ownership, including the opportunity to decide whether one wants to remove their data from databases and the rights to know about the process and purpose of digital tools.^31,46,67,68^ More importantly, some ethical and societal concerns have been raised, including the issue of disempowerment through increased control of others (social media followers and health promoters), the disintegration of state responsibility for health, and an excessive trust in lives represented only through numbers.^69,70^ However, we found that rather than having disempowering effects,^69^ self-tracking can also bring patients with thyroid chronic conditions “*hope*,” and some users indicated that “*it is reassuring and empowering to know there are some things in my control*.” mHealth may, therefore, represent a valuable step toward a patient-centered treatment approach for thyroid health. It can do so by (1) improving the health literacy of thyroid patients through accurate and accessible information, (2) offering support with navigating the complexities of the medical system, and (3) providing users with features for tracing a personal path toward their own health and well-being.

Although health care professionals can benefit from a facilitated diagnosis and disease management process,^18,19,22^ patients using mHealth can more easily partner with their physician to make shared decisions about their health. mHealth solutions such as the BOOST Thyroid app can also provide de-identified data sets and contribute to the advancement of research on thyroid physiology for a longer period of time in large samples of patients. In this way, an mHealth approach can be helpful even for people who opt out of using it: the results of this research may benefit the understanding and diagnosis of thyroid disease for all members of society and will help both patients and physicians alike to jointly search for the most appropriate combination of medical treatment and lifestyle interventions.

## Conclusion: looking toward the future of gender equality in health

Our study among users of an mHealth app for patients with thyroid chronic conditions, which sought to draw upon the positive experiences of thyroid patients in using mHealth, found that a digitalized approach may potentially (1) contribute to revolutionizing and speeding up medical research on thyroid conditions, (2) help creating and implementing successful self-management strategies for chronic conditions, and (3) save time for health care practitioners. Using mHealth, three major problems of today's management of chronic conditions can be improved: adherence to therapy—a major problem in the current treatment of chronic conditions, speed and accuracy of diagnostics, and patient empowerment.^71,72^ Digital tools, with their ability to quickly implement patient and doctor feedback in their product, may offer a novel more patient-centered solution for the care of chronic health problems.^72,73^ There remains a need for better and faster communication between health care practitioners and the digital tech world, for more research and tech world collaboration, and also, parenthetically, for more patients working in the tech industry to move this patient-centered agenda forward.

## Supplementary Material

Supplemental data

Supplemental data

Supplemental data

Supplemental data

Supplemental data

Supplemental data

Supplemental data

Supplemental data

## Abbreviations Used

BMI: body mass index
BMR: basal metabolic rate
GDPR: General Data Protection Regulation
mHealth: mobile health
