# Supplementary material for: Improving the Quality of Life of Patients with an Underactive Thyroid Through mHealth: A Patient-Centered Approach
Source: Womens Health Rep (New Rochelle). 2021 Jun 28;2(1):182–94. doi: 10.1089/whr.2021.0010 (PMC8243709; doi:10.1089/whr.2021.0010)
Supplement: Supplemental data [file Supp_FigureS1.docx]

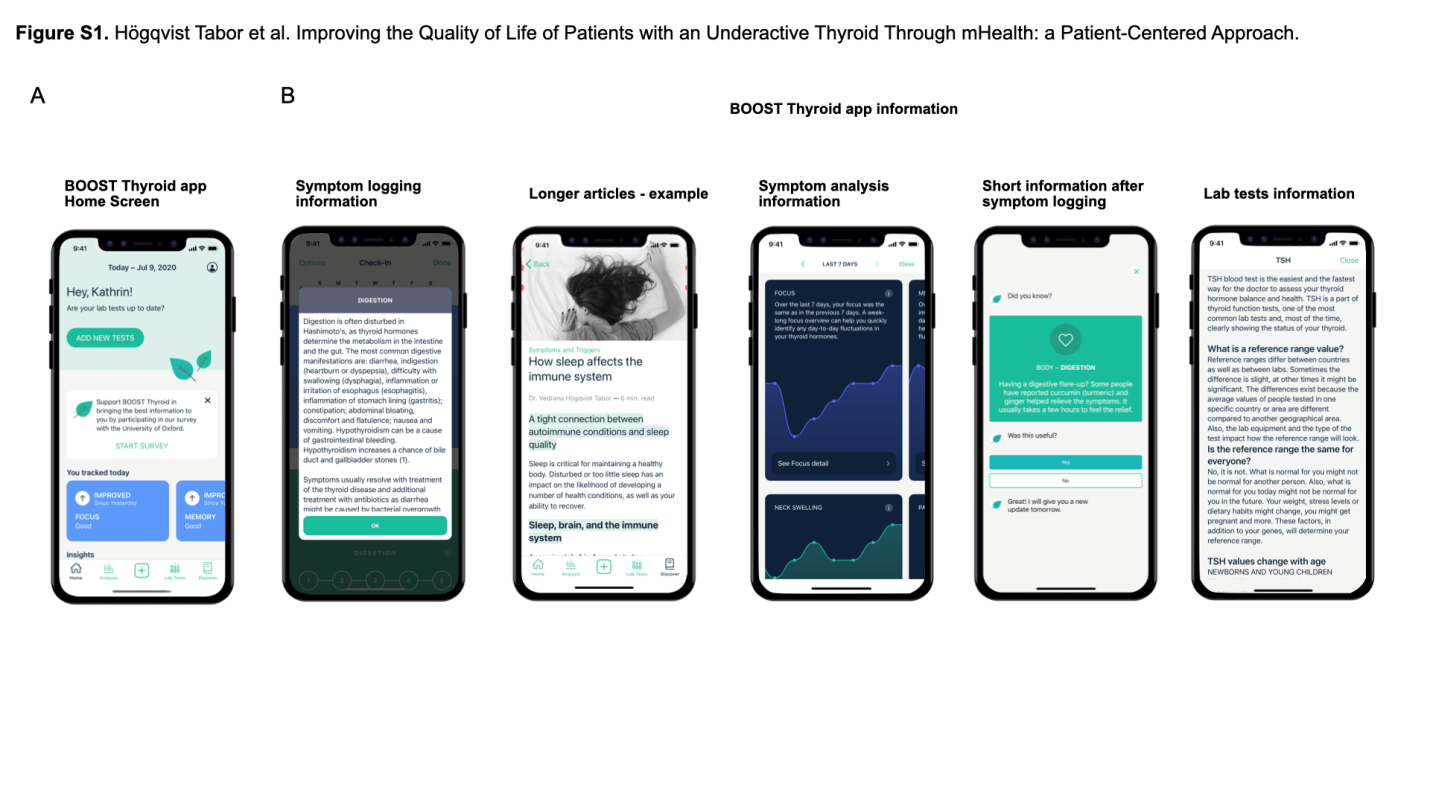


**Supplementary Figure 1.** BOOST Thyroid app user interface. **A.** BOOST Thyroid app *Home Screen*, where wellbeing survey was shown to app users; **B.** Examples of different types of in-app information shown to BOOST Thyroid app users (symptom logging information, longer articles, symptom analytics information, information after symptom logging and lab test information)
