# Supplementary material for: Improving the Quality of Life of Patients with an Underactive Thyroid Through mHealth: A Patient-Centered Approach
Source: Womens Health Rep (New Rochelle). 2021 Jun 28;2(1):182–94. doi: 10.1089/whr.2021.0010 (PMC8243709; doi:10.1089/whr.2021.0010)
Supplement: Supplemental data [file Supp_TableS2.docx]

Högqvist Tabor et al. Supplementary Table 2

| **Characteristics of hypothyroid patients** | **General population** | **BOOST**  **Thyroid users** | | | **BOOST**  **Thyroid survey respondents** | | | | | |
| --- | --- | --- | --- | --- | --- | --- | --- | --- | --- | --- |
| **Female - male ratio** | 10-1 {ref} | 7-1 | | | 13-1 | | | | | |
| **Age range (mean age)** | 35+ {ref} | 16-100 | | | 18- 76 | | | | | |
| **BMI** | NA | 25% 23.03  50% 26.60  75% 31.18 | | | 25% – 21.71  50% – 24.92 | | | | | |
|  |  |  |  |  |  | 75% – 30.12 SD | | | |  |
|  |  |  | SD 6.32 |  |  | – 6.58 |  | | | |
| **TSH (SD)** | NA | 25% – 0.96  50% – 2.22  75% – 4.36  SD – 8.36 | | | 25% – 0.56  50% – 1.51 | | | | | |
|  |  |  |  |  |  | 75% – 2.7 | | |  | |
|  |  |  |  |  |  | SD – 7.66 | |  | | |
