# Supplementary material for: Improving the Quality of Life of Patients with an Underactive Thyroid Through mHealth: A Patient-Centered Approach
Source: Womens Health Rep (New Rochelle). 2021 Jun 28;2(1):182–94. doi: 10.1089/whr.2021.0010 (PMC8243709; doi:10.1089/whr.2021.0010)
Supplement: Supplemental data [file Supp_FigureS2.docx]

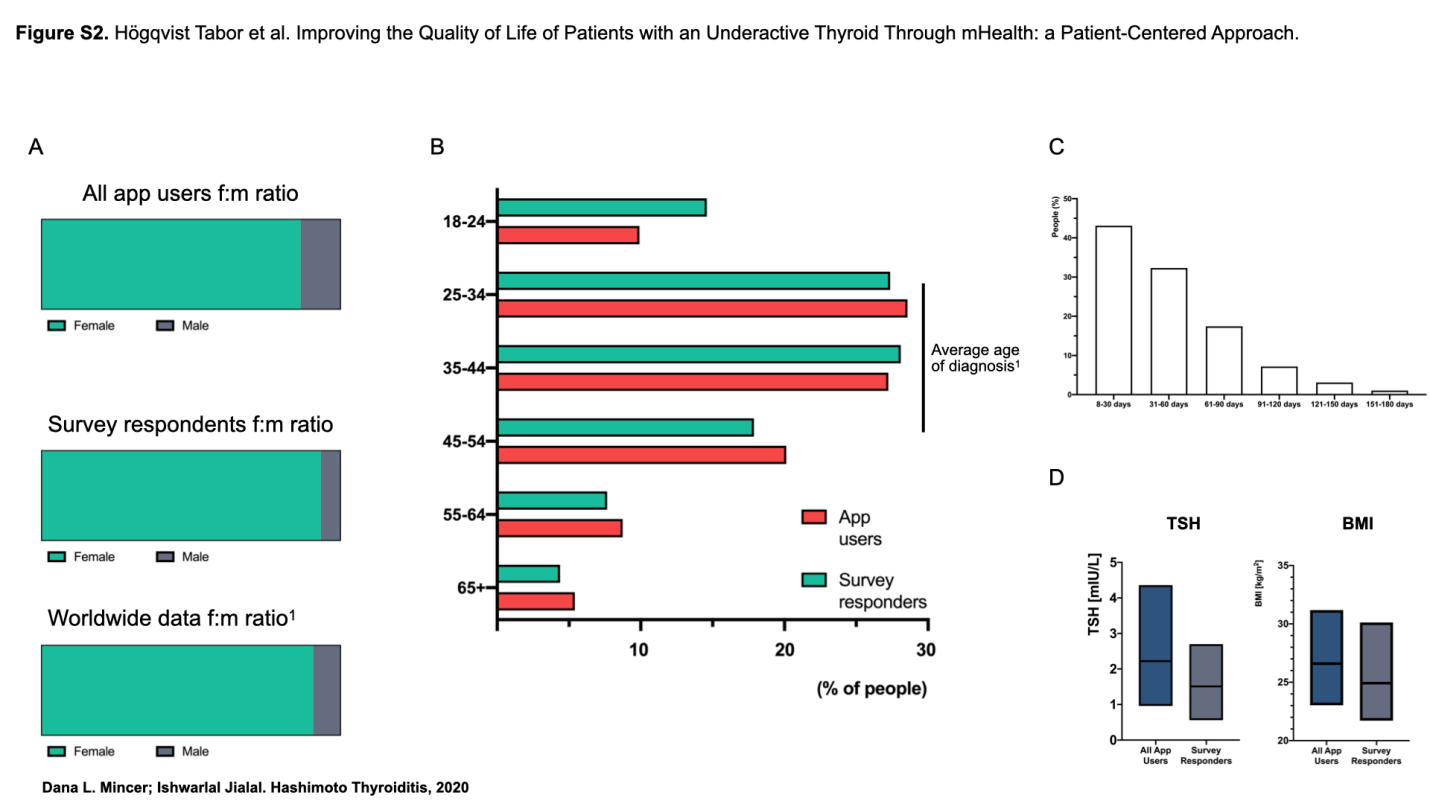


**Supplementary figure 2. Demographics of BOOST Thyroid app users and survey respondents. A.** Female to male ratio of an underactive thyroid reported in medical literature, overall female to male ratio of all the BOOST Thyroid app users, and the female to male ratio of survey respondents; **B.** Age distribution of BOOST Thyroid app users and survey respondents and indication of the average age of diagnosis as reported in the medical literature; **C.** Distribution of length of the BOOST Thyroid app usage for survey respondents; **D.** TSH (Thyroid Stimulating Hormone) value distribution in BOOST Thyroid app population and survey responders.
