# Supplementary material for: Improving the Quality of Life of Patients with an Underactive Thyroid Through mHealth: A Patient-Centered Approach
Source: Womens Health Rep (New Rochelle). 2021 Jun 28;2(1):182–94. doi: 10.1089/whr.2021.0010 (PMC8243709; doi:10.1089/whr.2021.0010)
Supplement: Supplemental data [file Supp_TableS1.docx]

Högqvist Tabor et al. Supplementary Table 1.

**Survey question**

| **Survey question** | **Question** | **Answer options** |  |
| --- | --- | --- | --- |
| **1** | Has the information you received through the BOOST Thyroid app been helpful to you in any way? | Yes No | Single answer |
| **2** | Which type of information was specifically useful to you? | Informational text describing every symptom, medication, and supplement in the daily check-in  Informational text describing lab tests  Informational text that appears after symptom check in Longer articles  Information on the analytics screen next to the graph  Other – please specify [open text] | Multiple answer |
| **3** | What information have you found the most helpful? | Information on symptoms Information on diet and exercise Information on medication Information on supplements Information on lab tests Information in surveys  Daily article  Daily information after symptom check-in | Single answer |
| **4** | How has this information been useful to you? | Helped me understand my body more Helped me understand my symptoms  Answered questions that medical doctors did not Helped me determine how to live healthier  Saved me time I would have spent on googling my symptoms  Other – please specify [open text] | Multiple answer |
| **5** | How has the information in the app impacted your doctor’s visits? | I have not visited the doctor since reading the information Visits are better structured  Visits are shorter Visits last longer  Visits are less frequent Visits are more frequent  Visits did not change changes  Other – please specify [open text] | Multiple answer |
| **6** | Has the information improved your health? | Yes  No | Single answer |

| **Survey question** | **Question** | **Answer options** |  |
| --- | --- | --- | --- |
| **7** | How has the information in the app improved your health? | Had less sick days  Visit doctor less frequently Less stressed  Sleeping better  Fewer symptoms in number Less symptoms in intensity  Other – please specify [open text] | Multiple answer |
| **8** | How would you describe your overall well-being before using BOOST Thyroid app? | Great Good OK  Not so good  Bad | Single answer |
| **9** | How did you manage your daily life and work before using the app | Very well OK  It was difficult to manage at times  It was difficult to manage most of the time  Other – please specify [open text] | Single answer |
| **10** | How easy was it for you to complete tasks ***before*** using the app? | I could easily complete tasks Tasks took longer to complete  Other – please specify [open text] | Single answer |
| **11** | How did you feel in general before using the app? | Happy Relaxed Rested Content Tired  Easily stressed Confused  Sad Frustrated  I had difficulty remembering things | Multiple answer |
| **12** | How would you describe your overall well-being ***after*** using the BOOST Thyroid app? | Great Good OK  Not so good  Bad | Single answer |

| **Survey question** | **Question** | **Answer options** |  |
| --- | --- | --- | --- |
| **13** | How have you been managing your life and work ***after*** using the app? | Very well OK  It was difficult to manage at times  It was difficult to manage most of the time  Other – please specify [open text] | Single answer |
| **14** | How easy is it for you to complete daily tasks after using the app? | I could easily complete tasks Tasks took longer to complete  Other – please specify [open text] | Single answer |
| **15** | How do you feel ***after*** using the app? | Happy Relaxed Rested Content Tired  Easily stressed Confused  Sad Frustrated  I had difficulty remembering things | Multiple answer |
| **16** | Can you tell us 3 ways the BOOST Thyroid app has influenced your health? | [open text] |  |
