# Supplementary material for: Improving the Quality of Life of Patients with an Underactive Thyroid Through mHealth: A Patient-Centered Approach
Source: Womens Health Rep (New Rochelle). 2021 Jun 28;2(1):182–94. doi: 10.1089/whr.2021.0010 (PMC8243709; doi:10.1089/whr.2021.0010)
Supplement: Supplemental data [file Supp_FigureS3.docx]

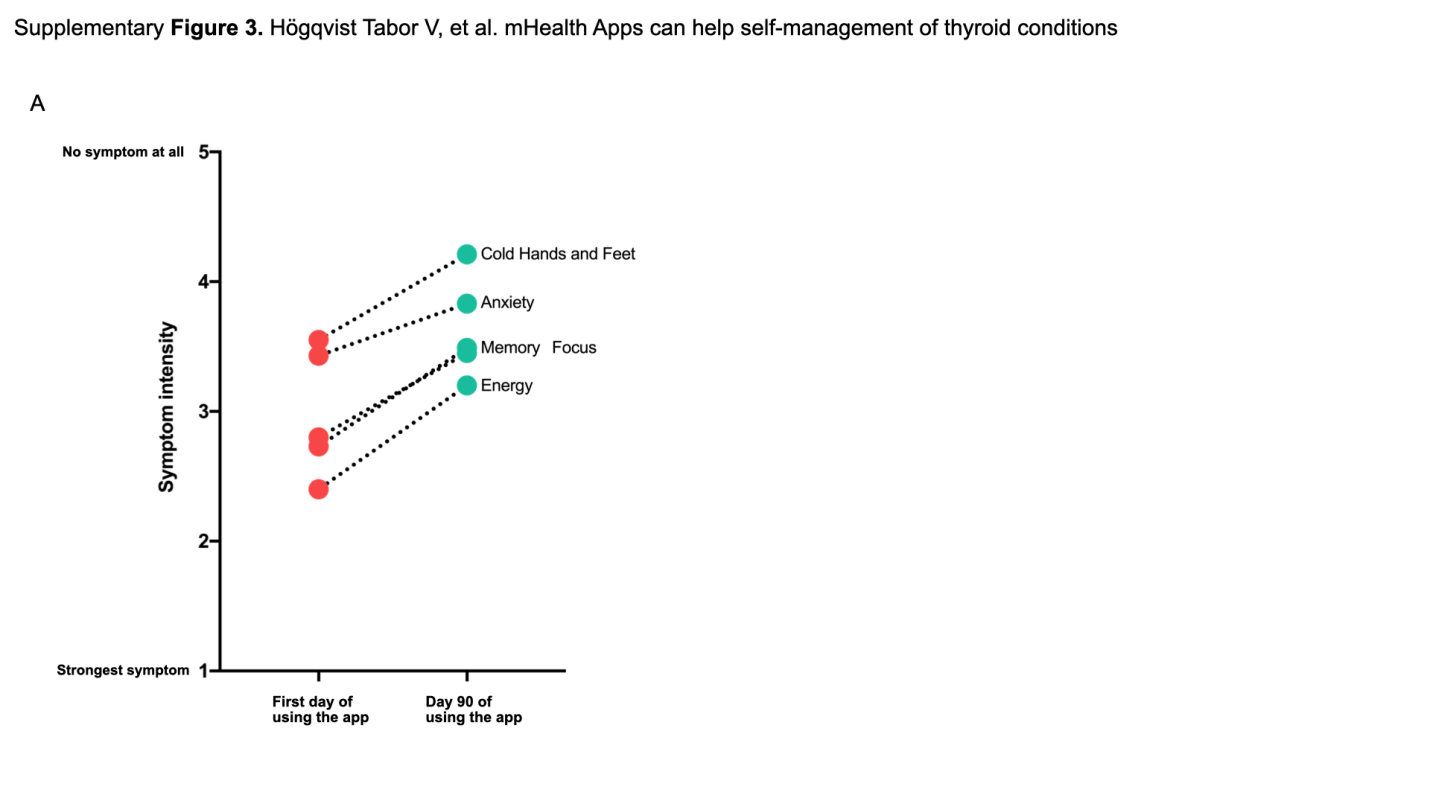


**Supplementary figure 3. Comparison of symptom experience of app’s users diagnosed with an underactive thyroid. A.** Five common symptoms experienced by patients with an underactive thyroid were assessed at day 0 (the first day user uses the app) and day 90 of app usage. Assessment of symptoms on a five-point intensity scale (5= no symptom at all, 1= the worst/strongest symptom)
