# Supplementary material for: Improving the Quality of Life of Patients with an Underactive Thyroid Through mHealth: A Patient-Centered Approach
Source: Womens Health Rep (New Rochelle). 2021 Jun 28;2(1):182–94. doi: 10.1089/whr.2021.0010 (PMC8243709; doi:10.1089/whr.2021.0010)
Supplement: Supplemental data [file Supp_TableS3.docx]

Högqvist Tabor et al. Supplementary table 3

| **Status of underactive thyroid patients** | **BEFORE APP** | **AFTER APP** |
| --- | --- | --- |
| **Wellbeing status** |  |  |
| Great (%) | 7 (2.5%) | 25 (8.9%) |
| Good (%) | 37 (13.2%) | 129 (45.9%) |
| OK (%) | 97(34.5%) | 104 (37%) |
| Not so good (%) | 113 (40.2%) | 23 (8.2%) |
| Bad (%) | 27 (9.6%) | 0 (0%) |
|  |  |  |
| **Ability to manage life- work balance** |  |  |
| Very well | 18 (6.8%) | 49 (18.6%) |
| OK | 75 (28.4%) | 157 (59.5%) |
| Difficult to manage at times | 120 (45.5%) | 53 (20.1%) |
| Difficult to manage most of the time | 51 (19.3%) | 5 (1.9%) |
|  |  |  |
| **Managing tasks** |  |  |
| Can easily complete tasks | 79 (36.6%) | 174 (80.6%) |
| Tasks took longer to complete | 137 (63.4%) | 42 (19.4%) |
|  |  |  |
| **General feeling** |  |  |
| Relaxed | 9 (3.4%) | 91 (33.8%) |
| Content | 19 (7.1%) | 146 (54.3%) |
| Happy | 23 (8.6%) | 61 (22.7%) |
| Confused | 103 (38.3%) | 25 (9.3%) |
| Sad | 107 (39.8%) | 27 (10%) |
| Problems with memory | 162 (60.2%) | 61 (22.7%) |
| Easily stressed | 170 (63.2%) | 56 (20.8%) |
| Frustrated | 173 (64.3%) | 44 (16.4%) |
| Tired | 215 (80%) | 93 (34.6%) |
