# Supplementary material for: Improving the Quality of Life of Patients with an Underactive Thyroid Through mHealth: A Patient-Centered Approach
Source: Womens Health Rep (New Rochelle). 2021 Jun 28;2(1):182–94. doi: 10.1089/whr.2021.0010 (PMC8243709; doi:10.1089/whr.2021.0010)
Supplement: Supplemental data [file Supp_TableS4.docx]

Högqvist Tabor et al. Supplementary Table 4

| **App impact** |  |
| --- | --- |
| **Positive health impact** |  |
| Helped understand own body more | 203 (57.9%) |
| Helped understand own symptoms | 219 (62.4%) |
| Answered questions that medical doctors did not | 148 (42.2%) |
| Helped determine how to live healthier | 161 (45.9%) |
| Saved time on googling symptoms | 103 (29.3%) |
| **Patient- doctor interaction** |  |
| I have not visited the doctor since reading the information | 151 (48.1%) |
| Visits are better structured | 67 (21.3%) |
| Visits are shorter | 7 (2.2%) |
| Visits last longer | 4 (1.3%) |
| Visits are less frequent | 16 (5.1%) |
| Visits are more frequent | 2 (0.64%) |
| Visits did not change | 65 (20.7%) |
| **Health benefit** |  |
| Less frequent dr visits | 17 (7.5%) |
| fewer sick days | 20 (8.8%) |
| Less symptoms | 42 (18.5%) |
| Better sleep quality | 47 (20.7%) |
| Less intense symptoms | 98 (43.2%) |
| Lower stress levels | 99 (43.6%) |
