# Supplementary material for: Improving the Quality of Life of Patients with an Underactive Thyroid Through mHealth: A Patient-Centered Approach
Source: Womens Health Rep (New Rochelle). 2021 Jun 28;2(1):182–94. doi: 10.1089/whr.2021.0010 (PMC8243709; doi:10.1089/whr.2021.0010)
Supplement: Supplemental data [file Supp_TableS5.docx]

Högqvist Tabor et al. Supplementary Table 5

**Themes**

| **Themes and reflections of underactive thyroid patients after using the BOOST Thyroid app** | | | | | | |
| --- | --- | --- | --- | --- | --- | --- |
| **Information is Empowering** | *“I understand my health circumstances better. It is reassuring and empowering to know there are some things in my control. The app has helped me be more consistent with supplements and exercise and reflects back my easy vs harder days…”* | *“I know now I’m not going crazy. I know there are things I can do to help outside of medication. I now know what to look for if medication needs adjusting…”* | *“I am just overall more aware of what the disease is and what to avoid to exacerbate issues…”* | *“It’s made me generally more aware of my choices and educated me about my thyroid disease…”* |  |  |
| **Tracking,** | *“This app has propelled me into making healthier decisions for my body and my condition. It made me realize my symptoms were thyroid related. With that information, I’ll be able to take better control of my health and get better health advice from my doctors…”* | *“Logging my daily symptoms has made me much more mindful of my physical and emotional experiences*  *-BOOST*  *motivates me to remain active in taking care of my thyroid health -BOOST motivates me to eat healthier…”* | *“Tracking, especially supplements intake, motivates me to keep up good habits. It*  *taught me lifestyle changes that have improved my overall well being a little…”* | *“ [I was] able to see trends arise from all the data collected, which better enabled me to get the correct medication dose, which I was struggling with for quite some time…”* | *“I knew I had to improve my eating and exercising habits, but the app pushed me to ACTUALLY DO IT…”* | *“I make sure I take all of my medication. I take better care of my health…”* |
| **Information** |  |  |  |  |  |  |
| **and Reminders** |  |  |  |  |  |  |
| **lead to better** |  |  |  |  |  |  |
| **care and** |  |  |  |  |  |  |
| **healthier** |  |  |  |  |  |  |
| **decisions** |  |  |  |  |  |  |
| **Support with** | *“Information about my test results to help me discuss symptoms, effects and reason why I need my medication increased even though it was being managed in the average range . Now it has been increased and I am feeling hugely better ver much like myself before I was diagnosed with this…”* | *“[ The app as ] Given me structure to improve my wellbeing as the medical system in my country refuses to give me access to the endocrinology department until I develop thyroid cancer. I am currently late stage Hashimoto’s with CVD at 35…”* | *"It allows me to have better conversations and follow-up questions with my doctors, and really has helped me to realize when I need to find better-trained doctors with up- to-date knowledge…”* |  |  |  |
| **the medical** |  |  |  |  |  |  |
| **system lead to** |  |  |  |  |  |  |
| **better** |  |  |  |  |  |  |
| **treatment** |  |  |  |  |  |  |

| **Themes and reflections of underactive thyroid patients after using the BOOST Thyroid app** | | | | | | |
| --- | --- | --- | --- | --- | --- | --- |
| **Improved health and well-being** | *“My mental health is improved because the app validates what I know about the disease from three generations of affliction and the published literature and that everything single endocrinologist I have ever consulted does not…”* | *“Information learned from this app has helped me to lose weight, make sure I’m getting the right nutrition and helped me to exercise in a way that doesn’t negatively impact me…”* | *“overall [the app] helps me know my symptoms aren’t me just crazy, they’re real…”* | *"I don’t feel so lonely with my illness…”* |  |  |
